# Supplementary material for: Effects of direction specific exercise training on athletic performance: a systematic review and meta-analysis
Source: PeerJ. 2024 Sep 16;12:e18047. doi: 10.7717/peerj.18047 (PMC11412225; doi:10.7717/peerj.18047)

Supplement 1. Effects of direction specific resistance and plyometric training on athletic performance outcomes: a systematic review and meta-analysis

Pubmed：442


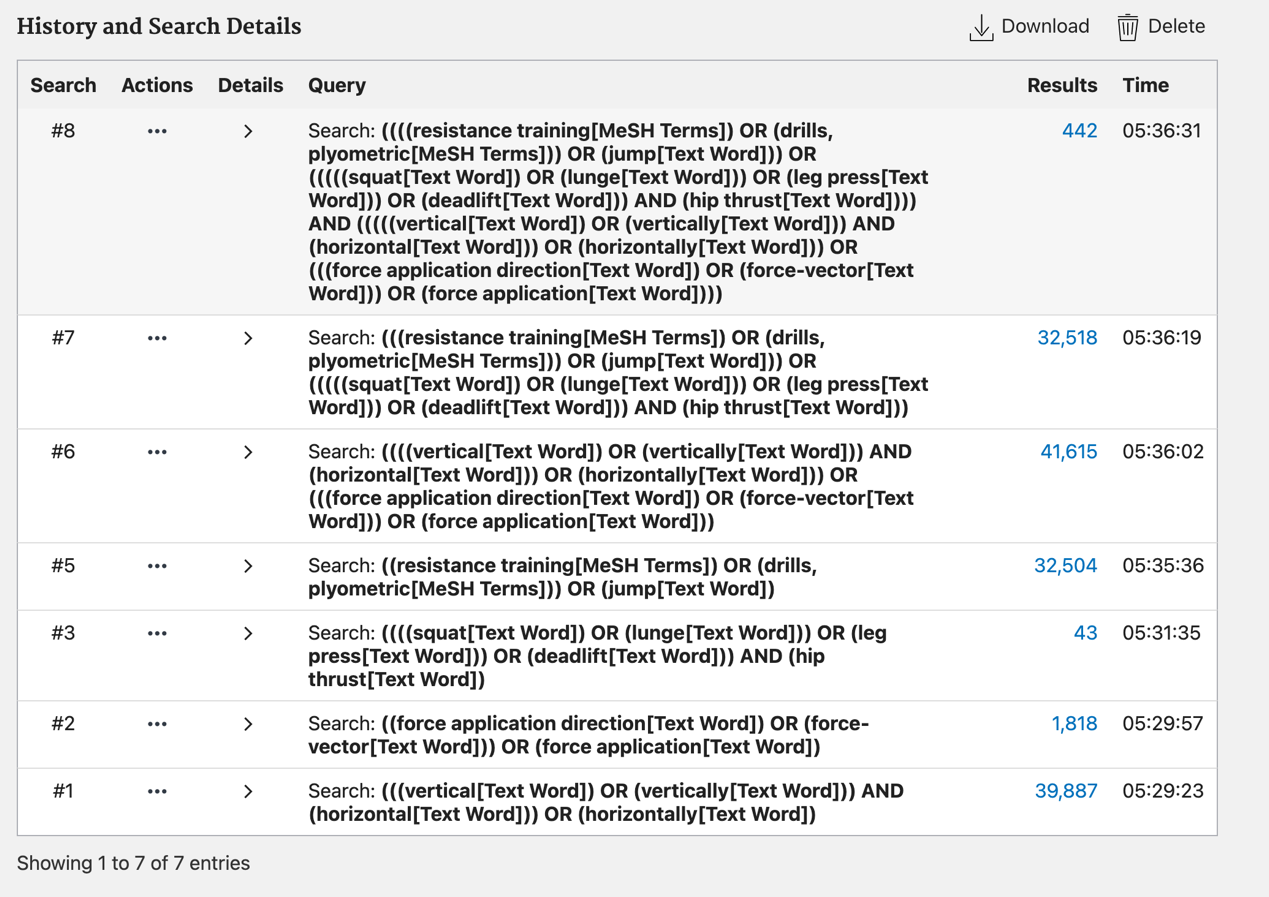


Wos: 1799


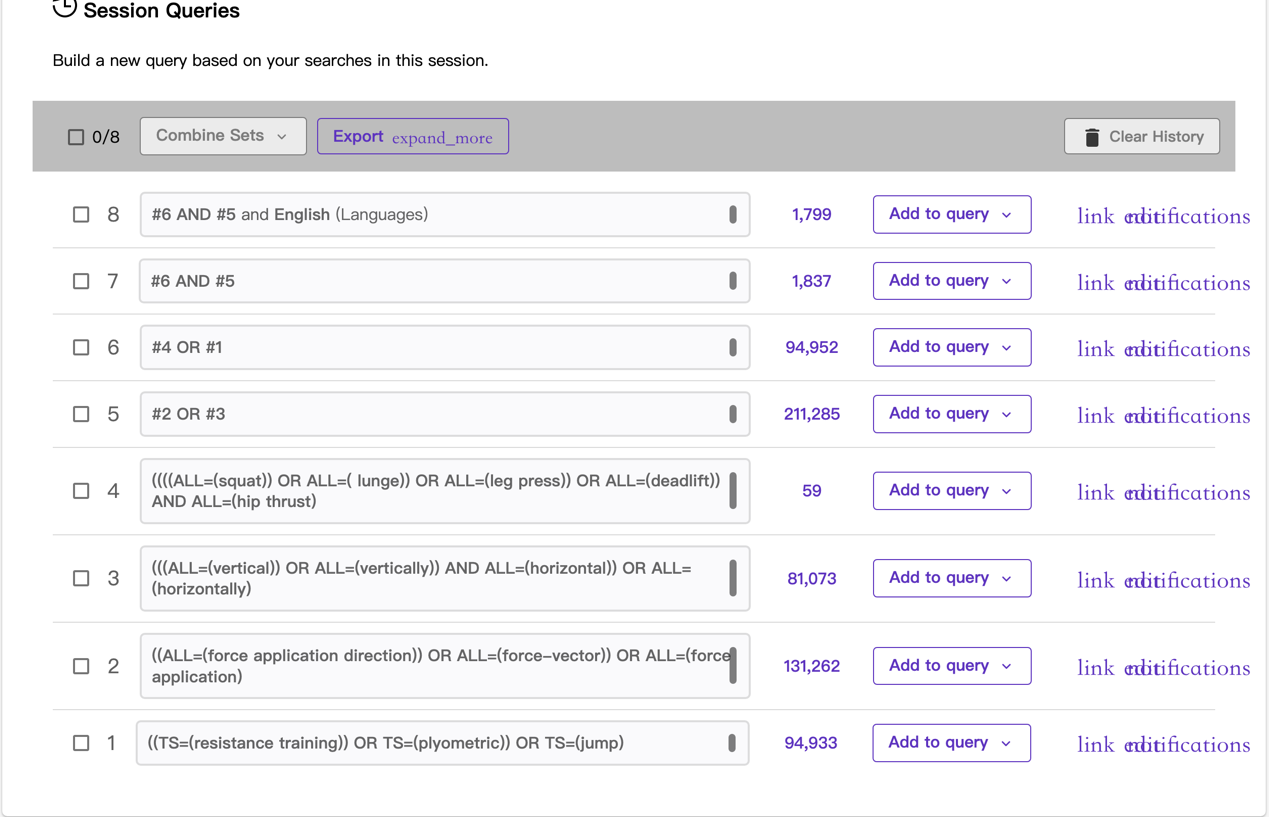


Medline：3375
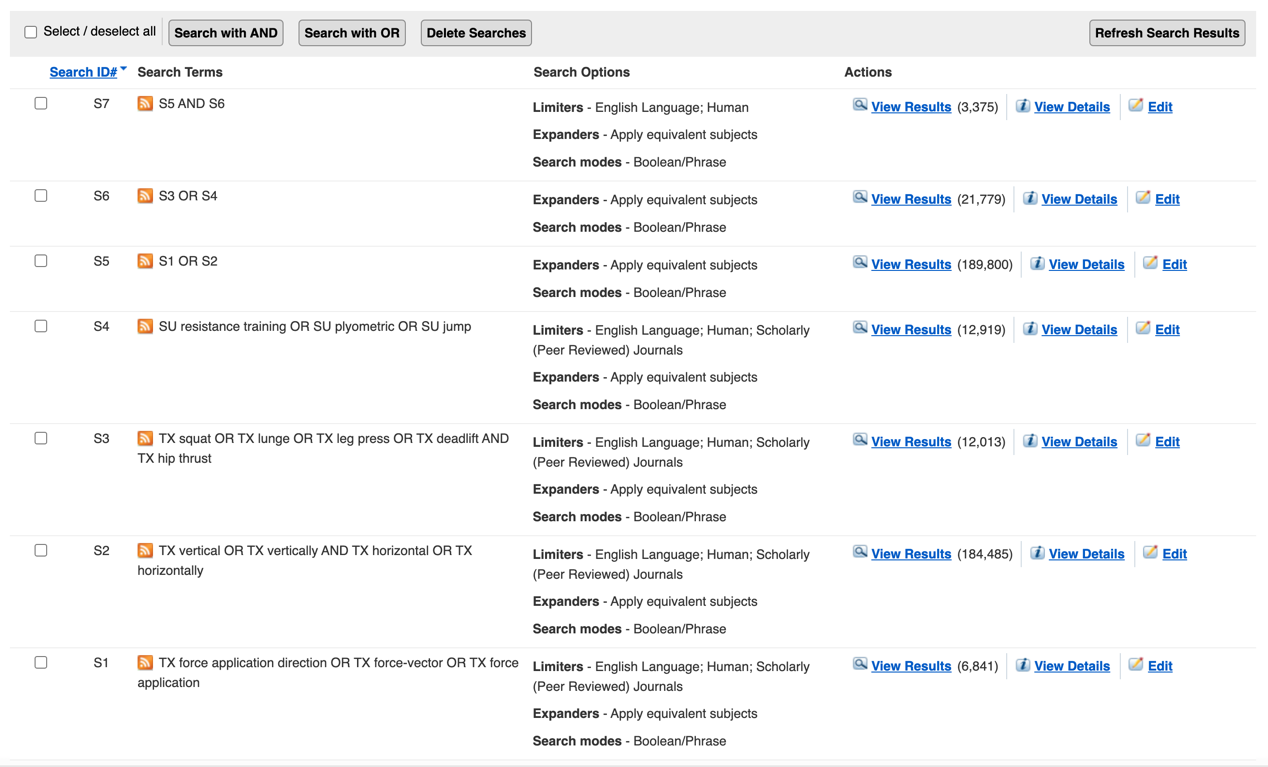

Supplement: Supplemental Information 2 [file peerj-12-18047-s002.docx]
